# Supplementary material for: Effect of foliar application of seaweed (organic fertilizer) on yield, essential oil and chemical composition of coriander
Source: PLoS One. 2022 Jun 2;17(6):e0269067. doi: 10.1371/journal.pone.0269067 (PMC9162348; doi:10.1371/journal.pone.0269067)
Supplement: Attachment — Submitted filename: Coriander (PLoS One)_rev.docx [file pone.0269067.s001.docx]

**Effect of seaweed organic fertilizer as foliar spray on yield,**

**essential oil and chemical composition of coriander**

Ayse Ozlem TURSUN^a^*

^a^University of Malatya Turgut Ozal, Battalgazi Vocational High School, Battalgazi, Malatya, Turkey

*Corresponding author; E-mail address: ayoztursun@hotmail.com

**Abstract**

The effect of fertilization on the yield of medicinal and aromatic plants is important. Among these fertilizers, seaweed, one of the organic fertilizers, is an environmentally friendly organic chemical. This study was conducted to determine the effects on the yield and essential oils of coriander, one of the oldest medicinal and aromatic plants, by applying different doses of seaweed as foliar before and at the beginning of flowering in 2 different locations. In this study, the essential oil content was found to be higher in the location with high sand content, and the yield and yield components were higher in location with low sand content and high organic matter content. Seaweed application at a dose of 2 ml L^-1^ showed positive effects on the development, yield and essential oil of coriander plant. Linalool (%) was determined as the most important essential oil component of the coriander plant. Foliar application of seaweed showed positive effects on the linalool ratio of the coriander plant. We can state that the foliar application of seaweed, which is one of the organic fertilizers, has important results in terms of increasing the production of the plant in coriander cultivation.

**Keywords**: Coriander, seaweed, linalool

**Introduction**

Organic fertilizers that provide basic and micronutrients have a really important role in soil quality (Palaniappan and Annudurai, 1999). Organic fertilizers for improving crop quality can be applied both from leaf and from soil.

Fertilizers that are applied on the leaf surface are treated on the leaf and stem by spraying, and these fertilizers are influenced to enter the plant from these plant organs. The advantages of foliar fertilizers are emerged more when there is limitation of soil fertilizers (Ver ma et al., 2000). In recent years, the importance of organic fertilizers or biostimulants are increased. Bio-organic matters or biostimulant matters that are known as microorganisms are used for improving nutrient intake, stimulating growth and rising plant tolerances and quality. The seaweed extracts that are considered as biostimulants has positive effects on plant development and growth (Hernandez-Herrera et al., 2014). In addition to improving plant growth, seaweeds are also used for increasing yield and quality, and, while they are also applied for obtaining products that are purged from chemicals (Spinelli et al., 2009). Due to being environmentally friendly, natural organic products, seaweed fertilizers are got quite popular and are used widely in agriculture (Khan et al., 2009). Liquid extracts which are obtained from seaweeds are recently used as foliar fertilizers on many plants (Crouch and Van Staden, 1994; Karthikeyan and Shanmugam, 2016). With the use of seaweed extracts for agricultural purposes, it was claimed that these extracts have positive effects on germination, root growth, leaf size, tolerating undesirable soil conditions and, nutrient intake from the soil (Hong et al.,1995). When the seaweed foliar fertilizers are applied by spraying on the plant leaves, they can increase cell division and growth speed and, these fertilizers can also be applied as liquid on the soil for cultivated plants. Seaweeds are rich resources of micro and macronutrients, amino acids, vitamins, which can affect positively cellular metabolism, plant growth, and yield (Khan et al., 2009). The most commonly used seaweed as a biostimulant is an algae riches in polysaccharides (alginate, fucoidan and laminarin), minerals, and vitamins, from North Atlantic Ocean and known as *Ascophyllum nodosum*. Besides, they are rich in bioactive compounds like polyphenols, lipids, and proteins (Holdt and Kraan, 2011). Chemical analysis of seaweeds and their extracts demonstrates that the presence of diversified plant growth regulators likes auxins and cytokines in various amounts (Panda et al., 2012). Furthermore, it is considered that extracts of seaweeds might be used instead of chemical fertilizers (Hernandez-Herrera et al., 2014).

Coriander (*Coriandrum sativum* L.) which belongs to the Apiaceae family, is one of the oldest medical plants that has been cultivated by humans (Laribi et al., 2015; Pooja et al., 2017). The coriander plant that originated from Mediterranean regions, it is mostly cultivated in South Asia countries, and also is cultivated in high mountain Middle East Europe and Southeast Africa countries (Ravi et al., 2007; Nadeem et al., 2013). The most used part of coriander plants are their fruits that has volatile oils are used in cosmetic, medicine, and spice industries, and its fresh and dry leaves are used as spice and vegetables, also its grounded mature fruits are directly used as spice (Gokduman and Telci, 2018). The volatile oils which are obtained from the fruits are used in food, alcohol, and cosmetic industries. Additionally, the volatile oils of coriander have antibacterial, antioxidant, antidiabetic, antidepressant, antifungal, antihypertensive, anticancer, improving memory, antimutagenic and diuretic effects (Momin et al., 2012; Wladyslaw and Nowak, 2015; Nguyen et al., 2020). There are 0.03-2.7 % volatile oils in matured coriander fruits. The most important component of volatile oil is linalool, and it takes around 50 to 70% in whole volatile oil compounds (Diederchsen, 1996; Telci et al., 2006a; Telci et al., 2006b; Telci and Hisil, 2008). Due to its valuable ingredient of linalool, coriander essential oil is used in the cosmetic industry. Genetic structures of volatile oil plants with climate conditions and agricultural practices can cause some changes in ingredients and components of volatile oil (Telci et al., 2006a; Telci et al., 2006b; Telci et al., 2010). The importance of chemical and organic fertilizers on the yield of medical and aromatic plants is reported in some studies (Kusuma et al., 2019; Suman et al., 2019; Ozyazıcı, 2021). The demand for the organic products has continuously increased in all over the world including Turkey. Because of this reason, uses of organic and microbial fertilizers have been spread all around the world. Yet, there are just a few studies about the effects of organic fertilizers on medical and aromatic plants in the literature. For this reason, this study was carried out to determine the changes on yield, quality and compounds of essential oils by applying in different doses and at different times (before or initial stage of flowering) of seaweed to two different coriander varieties. Moreover, in this study, changes in linalool, an important component of coriander essential oil, were determined according to seaweed application doses.

**Materials and Methods**

**Site description**

Field tests were set up at Malatya Turgut Ozal University Malatya/Turkey (39.01.51 °N, 38.29.15 °E). The setups were installed in two different zones which have two different soil structures in coriander production season of 2021 (Location A and Location B). The soil structures are shown in Table 1. When the test fields are compared, the soil type is sandy-clay loam and it is rich in organic nutrients in Location A but, in Location B, the soil type is sandy and the soil is poor in organic nutrients (Table 1).

**Table 1**. Physical and chemical properties of soils in experimental areas (Locations) (0-20 cm)

| Compounds | Locations | |
| --- | --- | --- |
|  | Location A | Location B |
| Sand (%) | 38 | 64 |
| Silt (%) | 22 | 10 |
| Clay (%) | 40 | 26 |
| pH | 8.6 | 8.7 |
| EC (dS m^-1^) | 0.3 | 0.2 |
| Organic matter (%) | 2.6 | 1.2 |
| CaCO_3_ (%) | 36.1 | 28.3 |
| P_2_O_5_ (kg ha^-1^) | 9.7 | 11.7 |
| K_2_O (kg ha^-1^) | 65.1 | 63.8 |

**Table 2.** Main climate data of the research site for the long term (1981–2019) and for the trial year.

|  | **Average temperature (^0^C)** | | **Total precipitation (mm)** | |
| --- | --- | --- | --- | --- |
| **Months** | **2021** | **1929-2020** | **2021** | **1929-2020** |
| May | 22.0 | 18.0 | 20.6 | 45.6 |
| June | 24.7 | 23.1 | 9.1 | 17.4 |
| July | 29.2 | 27.0 | 0.2 | 3.9 |
| August | 28.2 | 27.0 | 23.2 | 3.5 |
| September | 22.8 | 22.5 | 5.7 | 8.1 |
| **Avarage** | 25.4 | 23.5 | -- | -- |
| **Total** | -- | -- | 58.8 | 78.5 |

**Experimental Design**

Soil preparation was conducted according to local practices for coriander production. In both testing fields, soil was cultivated using a chisel plough, followed by a disk harrow and finally by a harrow to obtain a smooth seedbed before sowing the crop. Erbaa and Gamze coriander varieties were used in the experiments, and both varieties were sown on 29 May 2021, with 25 cm spacing between rows and 5 cm between plants. At the time of sowing, 50 kg ha^-1^ N and 50 kg ha^-1^ P_2_O_5_ were applied to the field. When coriander plants reached in during the stem elongation period, 25 kg N ha^-1^ was applied as urea nitrogen. Every plot has 4 lines and plot length were measured as 4 m. In both study fields, weed management were provided by traditional agricultural practices in case to be needed. There were 1m spaces between plot and block, the two middle lines in every plot were harvested and used for yield. Fields were irrigated with drip irrigation. After flowering period, the plants were not irrigated.

The tests were set up according to testing pattern and, main plots were consisted of coriander varieties (Erbaa and Gamze) and sub plots were consisted of seaweed fertilizer dosages. Seaweed fertilizer dosages were applied in two periods: 40 days after the sowing and at the 60^th^ day (starting of flowering period). The dosages were applied as liquid from the leaves as 0.5 mL^-1^, 1 mL^-1^ and 2 mL^-1^ and there were not any other applications in control plots. The seaweed applied fertilizer which has 10% organic compounds, 0.4% alginic acid, 9.69 ds/m EC, with 7.8-9.8 Ph, is in liquid form. At the end of the experiment, coriander plants were harvested when the seeds turn brown in 04 September 2021 and plant length (cm), crude protein percentage (%), seed yield (kg ha^-1^), volatile oil yields (L ha^-1^), volatile oil ratios and volatile oil nutrients were analysed. Volatile oil yields (L ha^-1^) were calculated with multiplication of detected percentage of volatile oil by obtained seed yields.

**Raw Protein Ratio**

Raw protein ratio (%) in plots was calculated according to the Dumas method. For this method, coriander samples were burned at 1200 ^0^C in an oven with oxygen gas and the burned samples get oxidized with organic elements. Then, the combusted gasses (O_2_, CO_2,_ H_2_O, N_2_ and nitrogen oxides NO_x_) were kept together and passes through traps. All the gases were eliminated excluding nitrogen and nitrogen oxide. Then, they were transformed into bonded nitrogen molecules or nitrogen oxides and they are carried into an oxidizer catalytic oven with a vector gas. Purification of combustion gases and dehydration, tungsten or copper compounds are applied to these gases for reduction reaction and all the nitrogen compounds are transformed into N_2_ forms. Detection is done by Thermal Conductivity Detector (TCD). A connected computer calculates the nitrogen concentration from the sample weight and with the signal of TCD. Content of crude protein is calculated by multiplication of measured nitrogen amount with by suitable factor (6.25) and it is presented by percentage (Tekkanat and Soylu, 2005).

**Isolation of essential oils**

The essential oil ratio was extracted using a 1 L Clevenger distillation apparatus. For this purpose, distilled water was used, and 50 g dried aerial parts were diluted with 500 ml distilled water (1:10 w/v) for 3 h for each application. Essential oil was dried over anhydrous sodium sulphate and stored in dark glass bottle at 4 °C until used in analyses.

The components of the essential oils of the plants were determined by gas-chromatographic method. Determination of essential oil components was carried out with Thermo Scientific ISQ Single Quadrupole model gas chromatographic device under the following conditions. TR-FAME MS model, 5% Phenyl Polysilphenylene siloxane, 0.25 mm inner diameter x 60 m length, 0.25 µm film thickness column was used. Helium (99.9%) was used as the carrier gas at a flow rate of 1 mL min^-1^. The ionization 22 energy was set at 70 eV, the mass range m/z was 1.2-1200 amu. Scan Mode was used for data collection. The MS transfer line temperature was 250 °C, the MS ionization temperature was 220°C, the injection port temperature was 220 °C, the column temperature was initially 50 ° C and the temperature was increased to 220 °C with a rate of heat increase of 3 °C min^-1^. The structure of each compound was identified using mass spectra with the Xcalibur program (Wiley 9) (Kara et al., 2020).

**Statistical analysis**

The data were underwent to statistical analysis. GLM model One way (ANOVA) analysis of variance was applied to evaluate the data in the studies of the effects of different seaweed applications and doses on coriander using IBM SPSS 25 statistical package program. The difference between treatments was found using the Duncan multiple comparison test (*p*<0.05).

**Results and Discussion**

Seaweed fertilizer was applied on the plants 40^th^ and 60^th^ days after sowing and it was applied with different dosages. Some significant increases were obtained in plant length, volatile oil percentage, volatile oil yield and crude protein percentage on two coriander varieties in different locations.

When the effects of the seaweed fertilizer considered, there were some significant differences on yield (p<0.001), plant length and protein percentage (p<0.01) and volatile oil percentage (p< 0.05) between the plants in different locations. However, there was not any important difference about yield of volatile oils (Table 3).

**Table 3.** Effects of seaweed applied at different times and doses in two different locations (± standard error)

|  | Yield (kg ha^-1^) | Plant height (cm) | Essential oil (%) | Essential oil yield (L ha^-1^) | Protein content (%) |
| --- | --- | --- | --- | --- | --- |
| Locations | | | | | |
| Location A | 1407.08±25.11a | 78.40±0.96a | 0.45±0.02a | 6.36±0.36 | 20.59±0.22a |
| Location B | 1137.88 ±16.17b | 65.70 ±0.53b | 0.66±0.04b | 7.56±0.45 | 19.03±0.25b |
| *F ration* | 524.59^***^ | 43.09^**^ | 19.57^*^ | 4.163^ns^ | 82.86^**^ |
| Variety | | | | | |
| Gamze | 1343.13 ±30.17a | 69.70 ±0.81a | 0.53±0.03a | 7.00 ±0.431 | 20.18±0.27a |
| Erbaa | 1201.83±23.25b | 74.40±1.48b | 0.58 ± 0.03b | 6.90±0.396 | 19.44±0.24b |
| *F ration* | 146.00^***^ | 7.85^*^ | 9.143^*^ | 0.30^ns^ | 28.17^**^ |
| Time application | | | | | |
| 40. days | 1312.50± 33.00a | 72.80 ±1.13 | 0.53±0.03 | 6.93±0.41 | 19.73±0.27 |
| 60. days | 1232.46 ± 22.49b | 71.30 ±1.28 | 0.57±0.04 | 6.99± 0.42 | 19.88±0.25 |
| *F ration* | 31.34^**^ | 5.56^ns^ | 2.34^ns^ | 0.64^ns^ | 1.87^ns^ |
| Seaweed doses (ml L^-1^) | | | | | |
| 2 | 1387.08±38.00a | 73.5±1.68a | 0.65± 0.05a | 8.82±0.60a | 21.19± 0.26a |
| 1 | 1326.25±38.90ab | 72.9± 1.81a | 0.67±0.04a | 8.72±0.43a | 20.40±0.23b |
| 0.5 | 1247.42±37.14b | 73.0±1.81a | 0.51±0.04b | 6.21±0.33b | 19.79±0.23b |
| Control | 1129.17±27.68c | 68.8±1.43b | 0.37±0.03c | 4.09±0.27c | 17.85± 0.34c |
| *F ration* | 112.83^***^ | 12.22^***^ | 25.24^***^ | 33.98^***^ | 42.68^***^ |

**p*< 0.05 (significant); ***p*< 0.01 (moderate significant); ****p*< 0.001 (highly significant); ns, non-significant. The difference between the averages represented with the same letter in the same column and same group is statistically insignificant.

When the coriander plants are compered in Location A, which has more clay and organic matter with low sand percentage, and the plants in Location B, which has less organic matter and clay with high sand percentage, it was claimed that in Location A plants the yield had increased 20%, the plant size had increased 16% and the protein amount had increased 7.5%. However, for Location B plants, there was opposite situation in volatile oil percentage and volatile oil yield (respectively about 72 and 16%) (Table 3). Effects of seaweed fertilizers, which had been applied in different times and with different dosages, on Gamze and Erbaa varieties in yield (p<0.001), protein percentage (p<0.01) and plant size were taken as significant differences. On the other hand, between the varieties, the difference in volatile oil yield was taken as not significant (Table 3).

The seaweed caused increase in yield, volatile oil yield and protein amount for Gamze variety but, for Erbaa variety, the seaweed caused increase in plant size and volatile oil. When the seaweed was applied at 40^th^ and 60^th^ days after the sowing, it had really significant effect for yield (p<0.01). Although, there were no effect for plant size, volatile oil yield and protein percentage. 8% increase in yield of coriander was obtained when the seaweed had been applied at the 40^th^ and 60^th^ days after sowing. It was claimed that when the seaweed is applied different at times and with different dosages to coriander plants, it has really important effects for yield, plant length, volatile oil percentage, volatile oil yield and protein percentages (p< 0.001). 2 ml L^-1^ seaweed application caused increase in all measurements when the plants compared with the control plants. It was claimed that with that application, coriander yield increased 19%, plant length increased 6.5%, volatile oil increased 43%, volatile oil yield increased 54% and protein percentage increased 16%. The seaweed that was applied from leaf caused positive effects on yield, plant length, volatile oil percentage, volatile oil yield and protein percentage (Table 3).

Table 4. Interactions of seaweed applied at different times and doses in two different locations (± standard error)

|  | | | Yield (kg ha^-1^) | Plant height (cm) | Essential oil (%) | Essential oil yield (L ha^-1^) | Protein content (%) |
| --- | --- | --- | --- | --- | --- | --- | --- |
| Varieties x Locations | | | | | | | |
| Gamze | Location A | | 1503.33±32.98a | 74.3±0.58 | 0.42±0.03 | 6.35±0.50 | 20.98±0.35 |
|  | Location B | | 1182.92±20.01c | 65.0±0.70 | 0.64± 0.05 | 7.67±0.69 | 19.38±0.35 |
| Erbaa | Location A | | 1310.83±26.15b | 82.6±1.40 | 0.48±0.04 | 6.36 ± 0.53 | 20.20±0.24 |
|  | Location B | | 1092.83 ±22.17d | 66.3±0.78 | 0.68± 0.02 | 7.45± 0.58 | 18.68±0.35 |
| *F ration* | | | 19.31^*^ | 4.19^ns^ | 0.57^ns^ | 0.34^ns^ | 0.07^ns^ |
| Time application x Locations | | | | | | | |
| 40. days | Location A | | 1472.92±39.25a | 78.80 ± 1.21 | 0.45±0.03 | 6.77±0.55ab | 20.49±0.30 |
|  | Location B | | 1152.08±25.95c | 66.80 ±0.81 | 0.61±0.05 | 7.08±0.63ab | 18.98± 0.38 |
| 60. days | Location A | | 1341.25±25.69b | 78.10 ±1.53 | 0.44± 0.03 | 5.94±0.46b | 20.69±0.32 |
|  | Location B | | 1123.67±109.44c | 64.5± 0.61 | 0.70± 0.05 | 8.04±0.63a | 19.08±0.33 |
| *F ration* | | | 13.04^**^ | 1.82^ns^ | 3.95^ns^ | 6.04^*^ | 0.25^ns^ |
| Locations x Seaweed doses | | | | | | | |
| Location A | | 2 | 1521.67±50.82a | 80.4±1.46 | 0.52± 0.05bcd | 7.85±0.77 | 22.00± 0.37a |
|  |  | 1 | 1470.83±46.82ab | 79.4±2.03 | 0.53±0.04bc | 7.66±0.61 | 20.75±0.31b |
|  |  | 0.5 | 1399.17±34.52b | 79.6±2.23 | 0.40± 0.03de | 5.60± 0.46 | 20.45±0.24b |
|  |  | Control | 1236.67± 25.62c | 74.3±1.59 | 0.35± 0.04e | 4.31±0.49 | 19.15±0.37c |
| Location B | | 2 | 1252.50±21.99c | 66.6±0.97 | 0.78± 0.07a | 9.78±0.88 | 20.38±0.16b |
|  |  | 1 | 1181.67±19.77cd | 66.3±1.35 | 0.83± 0.05a | 9.78±0.88 | 20.05±0.30b |
|  |  | 0.5 | 1095.67±19.73de | 66.5± 0.97 | 0.63± 0.05b | 6.32±0.42 | 19.13±0.30c |
|  |  | Control | 1021.67± 21.14e | 63.2±0.53 | 0.38± 0.03de | 3.88±0.25 | 16.55±0.20d |
| *F ration* | | | 3.42^**^ | 0.81^ns^ | 4.53^**^ | 2.22^ns^ | 3.3^*^ |
| Time application x Seaweed doses | | | | | | | |
| 40.days | | 2 | 1468.33±60.19a | 73.1± 2.00 | 0.58±0.06 | 8.53± 0.85 | 21.28± 0.34 |
|  |  | 1 | 1360.83±65.45ab | 75.1±2.32 | 0.70 ± 0.07 | 9.08 ± 0.65 | 20.35± 0.33 |
|  |  | 0.5 | 1291.67±59.52bc | 74.2±2.46 | 0.48 ± 0.04 | 6.00± 0.36 | 19.45±0.40 |
|  |  | Control | 1129.17± 40.03d | 68.8 ± 2.06 | 0.37 ± 0.04 | 4.09± 0.39 | 17.85 ± 0.49 |
| 60. days | | 2 | 1305.83± 39.21bc | 73.9±2.79 | 0.72 ± 0.08 | 9.10±0.88 | 21.10±0.40 |
|  |  | 1 | 1291.67±42.74bc | 70.7 ± 2.73 | 0.66±0.05 | 8.36±0.58 | 20.45±0.32 |
|  |  | 0.5 | 1203.17±42.67cd | 71.9±2.72 | 0.55 ± 0.06 | 4.09± 0.39 | 20.13 ± 0.22 |
|  |  | Control | 1129.167± 40.03d | 68.8±2.06 | 0.37 ± 0.04 | 4.09± 0.39 | 17.85 ± 0.49 |
| *F ration* | | | 10.16^***^ | 3.46^ns^ | 1.86^ns^ | 0.56^ns^ | 0.71^ns^ |

**p*< 0.05 (significant); ***p*< 0.01 (moderate significant); ****p*< 0.001 (highly significant); ns, non-significant. The difference between the averages represented with the same letter in the same column and same group is statistically insignificant.

The interactions of Locations x varieties (*p*< 0.05); Locations x Time application (*p*< 0.01); Locations x Seaweed doses (p< 0.01); Time application x Seaweed doses (p< 0.001); while volatile oil percentages (%) are Locations x Seaweed doses (*p*< 0.01) and Time application x Locations x Seaweed doses (*p*< 0.05), volatile oil percentage is Locations x Seaweed doses (*p*< 0.01); yield of volatile oil Location x Time application and for protein percentages Location x Seaweed doses (p< 0.05). Locations x Time application interaction; when the Gamze end Erbaa coriander varieties are cultivated in Location A (high clay ratio and high amount of organic matter), the yield increases. For Location x Time interaction, the yield of Gamze variety was higher (Table 4).

Locations x Time interaction: it was obtained that when the seaweed is applied at the 40^th^ day after sowing, the yield in Location A (high clay ratio and high amount of organic matter) had reached the highest value. The second highest yield was obtained from the seaweed application at the 60^th^ day after sowing in Location A. In Location B, the yield values were less when they are compared to Location A plants at the fertilizer application times (40^th^ and 60^th^ days). The highest volatile oil yield was obtained from the seaweed application at the 60^th^ day after sowing in Location B and, the second highest volatile oil yield was obtained from the seaweed application at the 40^th^ day in Location B (8.04 ± 0.63, 7.08 ± 0.63, respectively) (Table 4).

In the case of Location x Seaweed doses interaction; the application of seaweed fertilizer at different times and with different doses caused increase in yield, plant length and protein ratio in Location A when it is compared with Location B. However, it is not the same for volatile oil yield and volatile oil ratio. In the soil which the coriander plant is cultivated, it was found that the volatile oil yield and ratio is higher in Location B. Additionally, in both locations (Location A and Location B) when the doses of seaweed were increased, it caused increases in the measured values with positive effects. The highest yield was obtained from 2ml L^-1^ seaweed application in both locations (except volatile oil ratio) (Table 4).

Time application x Seaweed doses interactions: when the seaweed was applied, it caused significant differences for yield. The highest yield belongs to 2 ml L^-1^ seaweed dose application at the 40^th^ day after sowing; and, 1 ml L^-1^ application of seaweed has the second place for yield. When the seaweed is applied on coriander, it causes increase in yield independently from location and variety.

**Table 5.** Time application x Locations x Seaweed doses interactions of seaweed applied at different times and doses in two different locations (± standard error)

| Time application x Locations x Seaweed doses | | | Yield (kg ha^-1^) | Plant height (cm) | Essential oil (%) | Essential oil yield (L ha^-1^) | Protein content (%) |
| --- | --- | --- | --- | --- | --- | --- | --- |
| 40. days | Location A | 2 | 1635.00 ± 64.49a | 79.20±0.70 | 0.55±0.08cde | 8.68±1.192bcd | 21.95±0.53 |
|  |  | 1 | 1545.00 ± 63.18ab | 80.80±2.68 | 0.52 ± 0.06cde | 7.86±0.799cde | 20.60±0.39 |
|  |  | 0.5 | 1475.00 ± 35.94bc | 80.70±2.82 | 0.40±0.04e | 5.93±0.614efgh | 20.25± 0.40 |
|  |  | Control | 1236.67± 38.01ef | 74.30±2.36 | 0.35±0.06e | 4.31±0.719gh | 19.15±0.55 |
|  | Location B | 2 | 1301.67±25.87def | 67.00±1.51 | 0.62±0.10bcd | 8.08±1.307bcde | 20.60±0.25 |
|  |  | 1 | 1176.67±35.93fg | 69.30±1.82 | 0.88±0.08a | 10.30±0.787ab | 20.10±0.54 |
|  |  | 0.5 | 1108.33±32.60gh | 67.70±1.28 | 0.55±0.04cde | 6.08±0.451efgh | 18.65±0.53 |
|  |  | Control | 1021.67±31.35h | 63.20±0.79 | 0.38±0.040e | 3.88±0.367h | 16.55±029 |
| 60. days | Location A | 2 | 1408.33 ±45.49cd | 81.70±2.88 | 0.48±0.07de | 6.73±0.818defg | 22.05±0.57 |
|  |  | 1 | 1396.67± 56.67cd | 78.00 ± 3.18 | 0.53±0.06cde | 7.46±0.990cdef | 20.90±0.52 |
|  |  | 0.5 | 1323.33±40.63de | 78.50±3.66 | 0.40±0.06d | 5.27±0.717fgh | 20.65± 0.30 |
|  |  | Control | 1236.67±38.01ef | 74.30± 2.36 | 0.35±0.06e | 4.31±0.719gh | 19.15±0.55 |
|  | Location B | 2 | 1203.33± 22.16efg | 66.20± 1.35 | 0.95±0.04a | 11.47±0.715ab | 20.15±0.17 |
|  |  | 1 | 1186.67±20.44fg | 63.30±1.02 | 0.78±0.05ab | 9.25±0.448abc | 20.00± 0.33 |
|  |  | 0.5 | 1083.00±24.19gh | 65.30±1.38 | 0.70±0.06bc | 7.56±0.580cdef | 19.60± 0.15 |
|  |  | Control | 1021.67± 31.35h | 63.20±0.79 | 0.38± 0.04e | 3.88±0.367h | 16.55± 0.29 |
| *F ration* | | | 2.817* | 0.52^ns^ | 3.82^*^ | 3.34^*^ | 0.32^ns^ |

**p*< 0.05 (significant); ns, non-significant. The difference between the averages represented with the same letter in the same column and same group is statistically insignificant.

In testing, an interaction occurred between Time application x Location x Seaweed doses and the highest yield was obtained from the seaweed applications with different doses at the 40^th^ day after the sowing in Location A. At the 60^th^ day after sowing, seaweed application with different doses also caused increase in yield in Location A. In both locations and in both application times the highest yield had been obtained from 2 ml L^-1^ seaweed application. Even though, the seaweed application at the 40^th^ and at the 60^th^ days did not increase the yield much in Location B. But it caused increase in volatile oil ratio and volatile oil yield. In case of application of 1 ml L^-1^ seaweed after 40 days from sowing and 2 ml L^-1^ seaweed application 60 days after the sowing causes increase significantly in yield and ratio of volatile oil. In Location B (with low amount of organic matter and high sand ratio) the yield was lower than Location A but, yield and ratio of volatile oil was higher (Table 5).

In the study which was done in two different locations that has different soil structures, the positive effects of seaweed applications on coriander plants were found. Sand, clay and organic matter in the soil affect the volatile oil ratio and yield regardless of the variety of coriander plant. The soil which is sandy, and has low amount of clay and organic matter, increased the yield of volatile oil and ratio of volatile oil. Yet, the other soil type (rich in organic matter and clay, poor in sand) caused decrease in volatile oil yield and ratio. Yield of coriander plant can depend on climate, genetic, ecological conditions and variety (Toncer et al., 1998; Kaplan and Cosge Senkal, 2021). Yet, on the other hand, plant length can be affected by ecological conditions and traditional applications alongside the genotype features (Ozyazıcı, 2021). In some studies, yield of coriander changes between 532-2713 kg ha^-1^ (Kassu et al., 2018; Izgi, 2020; Ozyazıcı, 2021) and Ozel et al (2019) claimed that plant length was between 34.50-111.63. Moreover, volatile oil ratios can change between 0.13-1.6% (Anwar et al., 2011; Hadian et al., 2012; Inan et al., 2014; Ozyazıcı, 2021) and protein ratio changes between 14.0-19.34% (Kaya et al., 2000; Kandemir, 2010). The values of yield, plant length, yield and ratio of volatile oil that were studied in the literature were in the same line with our studies, while the protein ratios that we obtained were higher when compared with these studies. Besides, there were some differences in plant length, volatile oil yield and protein ratios between the coriander varieties but these differences might be caused by genotype, soil and climate conditions, chemical fertilizer, specie and quantity (Ozyazıcı, 2021).

In the study, increase of amount of sand in the soil caused increase in volatile oil ratio and yield (Location B). The results that were obtained from this study are similar to other studies. This situation is compatible with other studies which shows that soil stress conditions like high pH, drought, low nutrients, sand, salinity cause increase in volatile oil ratios (Jeshni et al., 2017; Bhatla 2018; Khalid and Ahmed, 2021). Likewise, Aboukhalid et al., (2017) reported that quality of volatile oils is affected by soil structure. Aziz et al., (2008) and Said-Al Ahl et al., (2019) were observed that volatile oil components ratio of *Thymus vulgaris* is higher when it is cultivated in loam soil than in clay soil. Khalid and Ahmed, (2021) had also similar results on Citrus plants. In this study, it was also claimed that when the sand amount increases, the volatile oil ratio and yield increases too.

Seaweed extracts can be applied from the leaves and also can be applied on soil. The yield increase on plants which was caused by seaweed fertilizer application is related with hormonal components especially cytokines that are present in seaweeds (Featonby-Smith and Van Staden, 1984). On the other hand, in different studies it is reported that when the seaweed extracts are applied from the leaves, the application affects root growth positively and as a result of this; plants can get more water and nutrients from the soil that is why the yield increases (Mancuso et al., 2006; Alam et al., 2013). In this study, plant length, protein and volatile oil ratio, yield and volatile oil yield increased because of seaweed application. The increases on coriander plant might be related with micro and macro nutrients, cytokines, auxins and betaines in seaweed extracts so, this speeds up photosynthetic period and helps vegetative growth (Devi and Mani, 2015). In previous studies, it was reported that seaweed caused increase in yields on other plants. Crouch and Van Staden, (1992) claimed that with seaweed yield on tomato plants increased 30% and, Basavaraja et al., (2018) stated that they got 26% increase in yield on hybrid corns. Also, in this study, when the seaweed had been applied under the suitable conditions (in Location A at the 40^th^ day, 2ml L^-1^) the yield increased about 25% (Table 5). Likewise in this study, with seaweed applications the yields were also increased in some plants like soybean plants (Rathore et al., 2009), strawberry (Roussos et al., 2009) or watermelon (Abdel-Mawgoud et al., 2010). Application of seaweed, an organic fertilizer, increases volatile oil ratios and this result was also claimed by Jhariya and Jain, (2017) on coriander, Garg, (2007) on fennel and Gharib et al., (2008) on marjoram; using organic and biofertilizers increase the ratio of volatile oil. Moreover, in this study it was claimed that by seaweed applications, increase in protein ratios obtained on coriander plants. In previous studies, it was determined that coriander protein ratios change between 11.3%-21.3% (Bayrak and Korkut, 1995; Kaya et al., 2000) and in this study the results are similar to previous studies. Especially, the seaweed caused a significant increase in protein ratios when the plants are compared with control group.

**Nutrients of Volatile Oils**

In this study, it was obtained that the coriander plants which are cultivated in two different locations have different amount of volatile oil compounds. In location A plants, there were 22 components and in Location B plants, there were 21 components. Linalool, α-Pinene, Geranyl Acetate and γ-terpinene were determined as highest components. Linalool, an important component of coriander, was obtained more in Location B which has more sandy soil structure. Among the varieties, more Linalool was obtained in Erbaa variety. Depending on the locations, there were some differences in the components; linalool ratios were 77.25%-87.29%, α-Pinene ratios were 2.03 %-5.66%, γ-terpinene ratios were 1.75%-4.28%, Geranly acetate ratios were 1.94%-5.81%. The seaweed application (2ml L^-1^) at the 40^th^ and at the 60^th^ days after sowing caused the highest amount of Linalool in Location A plants (for both Gamze and Erbaa varieties). Still, in both coriander varieties which had been applied seaweed have higher Linalool when they are compared with control group. In Location B, the highest linalool amount was taken in Gamze variety which had been applied 1 ml L^-1^at the 60^th^ day after sowing but, for Erbaa variety, the highest amount of linalool was taken from 2ml L^-1^ seaweed application at the 40^th^ and at the 60^th^ days after sowing. In different studies on coriander plants, linalool ratio changes were reported as; Delaquis et al., (2002) 69.80%, Ozel et al., (2009) 76.12% - 82.74%, Gucuk, (2014) 53% – 74%, Beyzi and Gunes, (2017) 83.81% - 91.40%. As Izgi, (2020) stated, the changes in linalool ratios might be related with ecological, traditional and variety differences. In the study, the high linalool ratio is related with seaweed application. Ozyazıcı, (2021) reported that organomineral and organic fertilizers increases linalool ratio.

**Table 6.** Essential oil ratios of different seaweed doses in coriander varieties at different periods (day 40 and day 60) in Location A.

|  | Varieties | | | Gamze | | | | | | | Erbaa | | | | | | |
| --- | --- | --- | --- | --- | --- | --- | --- | --- | --- | --- | --- | --- | --- | --- | --- | --- | --- |
|  | Time application | | | 40.days | | | 60. days | | |  | 40. days | | | 60. days | | |  |
|  | Seaweed doses (ml L^-1^) | | | 2 | 1 | 0.5 | 2 | 1 | 0.5 | Control | 2 | 1 | 0.5 | 2 | 1 | 0.5 | Control |
| RT | Compound Name | SI | RSI |  |  |  |  |  |  |  |  |  |  |  |  |  |  |
| ***6.57*** | ***α-Pinene*** | ***990*** | ***994*** | ***2.04*** | ***5.66*** | ***3.98*** | ***3.28*** | ***3.92*** | ***2.09*** | ***3.83*** | ***4.37*** | ***2.60*** | ***4.20*** | ***3.85*** | ***2.55*** | ***4.19*** | ***3.28*** |
| 7.58 | Camphene | 988 | 991 | - | - | 0.24 | - | - | - | 0.21 | - | - | - | 0.33 | 0.12 | 0.20 | - |
| 7.57 | β-Pinene | 776 | 788 | 0.49 | 0.42 | 0.31 | 0.31 | 0.28 | 0.14 | 0.28 | 0.28 | 0.20 | 0.29 | 0.45 | 0.21 | 0.30 | 0.25 |
| 8.69 | β-phellandrene | 974 | 986 | 0.10 | 0.13 | 0.12 | 0.10 | 0.11 | 0.06 | 0.11 | 0.11 | 0.08 | 0.11 | 0.16 | 0.08 | 0.11 | 0.09 |
| 8.99 | β-myrcene | 986 | 994 | 0.29 | 0.33 | 0.37 | 0.29 | 0.32 | 0.20 | 0.32 | 0.26 | 0.24 | 0.31 | 0.36 | 0.25 | 0.28 | 0.22 |
| 10.03 | Limonene | 990 | 994 | 0.68 | 0.78 | 0.81 | 0.65 | 0.70 | 0.48 | 0.71 | 0.64 | 0.56 | 0.70 | 0.66 | 0.53 | 0.64 | 0.56 |
| ***11.10*** | ***γ-terpinene*** | ***995*** | ***995*** | ***1.75*** | ***4.24*** | ***4.28*** | ***2.65*** | ***3.97*** | ***2.79*** | ***3.83*** | ***3.23*** | ***3.18*** | ***3.78*** | ***3.46*** | ***3.00*** | ***3.68*** | ***3.25*** |
| 11.98 | Terpinolene | 915 | 931 | - | 0.25 | 0.24 | 0.21 | 0.24 | 0.16 | 0.20 | 0.18 | 0.17 | 0.21 | 0.23 | 0.15 | 0.18 | 0.16 |
| 12.00 | Eucalyptol | 909 | 919 | 0.69 | - | - | - | - | - | - | - | - | - | - | - | - | - |
| 12.68 | Cymene | 975 | 993 | 0.65 | 1.09 | 1.08 | 0.76 | 0.88 | 0.58 | 0.98 | 0.91 | 0.81 | 1.09 | 0.70 | 0.80 | 1.11 | 0.83 |
| ***20.63*** | ***Linalool*** | ***997*** | ***998*** | ***82.03*** | ***77.25*** | ***76.39*** | ***83.18*** | ***80.43*** | ***80.18*** | ***78.01*** | ***82.61*** | ***84.98*** | ***80.80*** | ***83.35*** | ***82.93*** | ***80.70*** | ***80.74*** |
| 22.81 | Decanal | 984 | 993 | 0.13 | 0.23 | 0.26 | 0.21 | 0.19 | 0.13 | 0.17 | 0.12 | 0.14 | 0.22 | 0.24 | 0.37 | 0.20 | 0.27 |
| 24.26 | Terpinen-4-ol | 977 | 985 | 0.16 | 0.07 | 0.14 | 0.09 | 0.10 | 0.12 | 0.13 | 0.08 | 0.13 | 0.09 | 0.09 | 0.16 | 0.11 | 0.11 |
| 25.94 | Camphor | 984 | 990 | 1.71 | 1.39 | 2.01 | 1.64 | 1.64 | 1.72 | 1.95 | 1.52 | 1.81 | 1.51 | 1.75 |  | 1.87 | 1.73 |
| 27.04 | α-Terpineol | 900 | 981 | 0.41 | 0.10 | 0.23 | 0.17 | 0.17 | 0.18 | 0.18 | 0.12 | 0.17 | 0.13 | 0.11 | 0.23 | 0.16 | 0.14 |
| 27.51 | Borneol | 969 | 984 | 0.31 | - | 0.11 | - | - | 0.09 | 0.10 | - | 0.09 | 0.07 | - | - | - | 0.09 |
| 27.56 | Myrtenylacetate | 726 | 754 | - | 0.06 | - | 0.06 | - | - | - | 0.06 | - | - | 0.10 | - | - | - |
| ***28.54*** | ***GeranylAcetate*** | ***988*** | ***988*** | ***1.94*** | ***4.38*** | ***4.15*** | ***2.90*** | ***3.64*** | ***2.49*** | ***3.56*** | ***3.68*** | ***3.04*** | ***3.60*** | ***2.54*** | ***4.35*** | ***4.32*** | ***3.45*** |
| 28.77 | trans-2-Decenal | 904 | 949 | 0.03 | 0.07 | 0.14 | 0.06 | 0.04 | 0.02 | 0.03 | 0.09 | 0.10 | 0.17 | 0.15 | 0.22 | 0.09 | 0.23 |
| 30.36 | Geranıol | 980 | 983 | 0.62 | 0.25 | 0.63 | 0.57 | 0.57 | 0.58 | 0.67 | 0.54 | 0.71 | 0.40 | 0.36 | 0.91 | 0.76 | 0.66 |
| 35.22 | 2-Dodecenal | 959 | 975 | 0.14 | 0.22 | 0.32 | 0.18 | 0.18 | 0.09 | 0.14 | 0.20 | 0.17 | 0.23 | 0.37 | 0.26 | 0.22 | 0.27 |
| 36.80 | Methylcinnamate | 990 | 992 | 3.88 | 1.13 | 3.37 | 1.80 | 1.59 | 2.25 | 0.02 | 0.10 | 0.17 | 1.19 | 0.02 | 0.07 | 0.19 | 0.02 |
|  | Others |  |  | 1.6 | 0.73 | 0.72 | 0.55 | 0.58 | 0.41 | 0.38 | 0.47 | 0.34 | 0.75 | 7.39 | 1.25 | 0.03 | 0.18 |
|  | Total | |  | 99.65 | 98.8 | 99.9 | 99.66 | 99.55 | 94.76 | 95.81 | 99.57 | 99.74 | 99.85 | 99.67 | 98.44 | 99.34 | 96.53 |

Tablo 7. Essential oil ratios of different seaweed doses in coriander varieties at different periods (day 40 and day 60) in Location B.

|  | Varieties | | | Gamze | | | | | | | Erbaa | | | | | | |
| --- | --- | --- | --- | --- | --- | --- | --- | --- | --- | --- | --- | --- | --- | --- | --- | --- | --- |
|  | Time application | | | 40. days | | | 60. days | | | | 40. days | | | 60. days | | |  |
|  | Seaweed doses (ml L^-1^) | | | 2 | 1 | 0.5 | 2 | 1 | 0.5 | Control | 2 | 1 | 0.5 | 2 | 1 | 0.5 | Control |
| RT | Compound Name | SI | RSI |  |  |  |  |  |  |  |  |  |  |  |  |  |  |
| ***6.57*** | ***α-Pinene*** | ***993*** | ***995*** | ***5.13*** | ***4.84*** | ***4.68*** | ***3.17*** | ***3.33*** | ***2.65*** | ***3.27*** | ***2.03*** | ***2.48*** | ***5.40*** | ***2.15*** | ***2.70*** | ***3.17*** | ***2.36*** |
| 7.56 | Camphene | 981 | 986 | 0.25 | 0.28 | 0.27 | - | - | - | - | 0.19 | - | 0.30 | - | - | - | - |
| 8.39 | β-Pinene | 971 | 985 | 0.37 | 0.35 | 0.38 | 0.43 | 0.29 | 0.21 | 0.23 | 0.28 | 0.19 | 0.38 | 0.17 | 0.20 | 0.21 | 0.16 |
| 8.69 | β-phellandrene | 972 | 975 | 0.13 | 0.12 | 0.11 | 0.09 | 0.09 | 0.07 | 0.08 | 0.11 | 0.08 | 0.14 | 0.07 | 0.08 | 0.09 | 0.08 |
| 8.99 | β-myrcene | 982 | 994 | 0.35 | 0.33 | 0.35 | 0.28 | 0.26 | 0.22 | 0.25 | 0.29 | 0.20 | 0.40 | 0.16 | 0.21 | 0.23 | 0.17 |
| 10.02 | Limonene | 988 | 995 | 0.88 | 0.87 | 0.87 | 0.64 | 0.61 | 0.52 | 0.66 | 0.69 | 0.51 | 0.95 | 0.40 | 0.55 | 0.58 | 0.42 |
| ***11.09*** | ***γ-terpinene*** | ***994*** | ***995*** | ***3.22*** | ***3.66*** | ***3.72*** | ***3.07*** | ***2.54*** | ***2.37*** | ***2.57*** | ***2.53*** | ***2.91*** | ***4.28*** | ***2.29*** | ***2.93*** | ***3.41*** | ***2.67*** |
| 11.95 | Terpinolene | 941 | 976 | 0.19 | 0.21 | 0.22 | 0.17 | 0.18 | 0.14 | 0.16 | 0.18 | 0.15 | 0.22 | 0.12 | 0.15 | 0.16 | 0.12 |
| 12.65 | Cymene | 985 | 991 | 1.03 | 1.91 | 2.05 | 0.88 | 0.78 | 0.72 | 1.56 | 0.33 | 0.77 | 1.79 | 0.75 | 1.02 | 0.96 | 0.76 |
| 18.76 | Linalooloxide | 879 | 924 | 0.12 | 0.03 | 0.07 | 0.05 | 0.04 | 0.02 | 0.09 | 0.05 | 0.02 | 0.09 | 0.04 | 0.05 | 0.06 | 0.02 |
| ***20.58*** | ***Linalool*** | ***996*** | ***997*** | ***80.15*** | ***79.52*** | ***77.26*** | ***83.48*** | ***84.31*** | ***81.73*** | ***78.73*** | ***87.15*** | ***85.88*** | ***78.81*** | ***87.29*** | ***85.25*** | ***83.72*** | ***81.49*** |
| 22.78 | Decanal | 991 | 997 | 0.35 | 0.30 | 0.38 | 0.28 | 0.34 | 0.26 | 0.34 | 0.26 | 0.18 | 0.28 | 0.22 | 0.21 | 0.24 | 0.17 |
| 24.25 | Terpinen-4-ol | 959 | 965 | 0.09 | 0.08 | 0.08 | 0.12 | 0.13 | 0.09 | 0.09 | 0.11 | 0.11 | 0.10 | 0.09 | 0.11 | 0.11 | 0.09 |
| 25.90 | Camphor | 984 | 989 | 1.60 | 1.42 | 1.61 | 1.66 | 1.49 | 1.47 | 1.36 | 1.71 | 1.65 | 1.64 | 1.49 | 1.71 | 1.63 | 1.43 |
| 27.06 | α-Terpineol | 963 | 980 | 0.13 | 0.08 | 0.10 | 0.17 | 0.16 | 0.15 | 0.12 | 0.12 | 0.15 | 0.12 | 0.14 | 0.16 | 0.15 | 0.13 |
| 27.52 | Borneol | 831 | 875 | 0.09 | 0.09 | - | 0.09 | 0.11 | 0.09 | 0.10 | 0.09 | 0.06 | 0.09 | 0.07 | 0.09 | 0.06 | 0.04 |
| ***27.72*** | ***Geranylacetate*** | ***869*** | ***924*** | ***3.42*** | ***4.27*** | ***5.81*** | ***3.64*** | ***3.57*** | ***3.53*** | ***3.89*** | ***2.25*** | ***2.86*** | ***3.43*** | ***2.75*** | ***2.83*** | ***3.37*** | ***2.40*** |
| 27.96 | 1-Decanol | 891 | 918 | 0.13 | 0.08 | 0.12 | - | 0.13 | - | 0.12 | 0.07 | - | 0.10 | 0.07 | - | - | - |
| 28.77 | trans-2-Decenal | 904 | 949 | 0.18 | 0.23 | 0.29 | 0.10 | 0.14 | 0.17 | 0.24 | 0.12 | 0.09 | 0.17 | 0.11 | 0.13 | 0.12 | 0.10 |
| 30.36 | Geranıol | 980 | 983 | 0.43 | 0.29 | 0.41 | 0.68 | 0.65 | 0.62 | 0.41 | 0.55 | 0.71 | 0.43 | 0.68 | 0.64 | 0.71 | 0.61 |
| 35.22 | 2-Dodecenal | 959 | 975 | 0.48 | 0.43 | 0.46 | 0.28 | 0.33 | 0.42 | 0.35 | 0.23 | 0.26 | 0.31 | 0.27 | 0.29 | 0.36 | 0.27 |
|  | Others |  |  | 0.53 | 0.46 | 0.62 | 0.56 | 0.46 | 0.86 | 0.60 | 5.41 | 0.38 | 0.43 | 0.41 | 0.59 | 0.40 | 1.29 |
|  | Total |  |  | 99.25 | 99.85 | 99.86 | 99.84 | 99.94 | 96.01 | 95.22 | 99.75 | 99.64 | 99.86 | 99.74 | 99.90 | 99.74 | 94.78 |

**Conclusion**

In this study, effects of seaweed on yield, protein ratio, volatile oil ratio, volatile oil yield of coriander plants (two different varieties) which are cultivated in two different locationswere analysed. As result, it was found that amount of coriander volatile oil was more in sandy soil. And, it was also found that seaweed applications have significant effects on yield, yield components and volatile oil components. Among the varieties, more yield was obtained from Gamze variety. The highest yield was obtained from 2ml L^-1^ seaweed application at the 40^th^ day after sowing in Location A which has mora clay and organic matter in the soil. The highest volatile oil ratio and yield was obtained from 2ml L^-1^ seaweed application at the 60^th^ day wafer sowing in Location B which has sandy soil. This study demonstrates that seaweed treatments as an organic fertilizer utilized on foliar area applied on coriander crop has significant results in terms of the importance for increasing the production.

**References**

Abdel-Mawgoud, A., Tantaway, A., Hafez, M.M. & Habib, H.A. (2010) Seaweed extract improves growth, yield and quality of different watermelon hybrids. *Research Journal of Agriculture and Biological Sciences*, 6(2), 161-168.

Aboukhalid, K., Alfaiz, C., Douaik, A., Bakha, M., Kursa, K., Agacka-Moldoch, M., Machon, N., Tomi, F. & Lamiri, A. (2017) Influence of environmental factors on essential oil variability in O. compactum BENTH growing wild in Morocco. *Chem. Biodivers*, 1-17, e1700158.

Alam, M.Z., Braun, G., Norrie, J. & Hodges, D.M. (2013) Effect of *Ascophyllum* extract application on plant growth, fruit yield and soil microbial communities of strawberry. *Canadian Journal of Plant Sci*ence, 93, 23–36.

Anwar, F., Sulman, M., Hussain, A.I., Saari, N., Iqbal, S. & Rashid, U. (2011) Physicochemical composition of hydro-distilled essential oil from coriander (Coriandrum sativum L.) seeds cultivated in Pakistan. *Journal of Medicinal Plants Researchs*, 5(15), 3537-3544.

Aziz, E.E., Hendawy, S.F., Ezz El-Din, A.A. & Omer, E.A. (2008) Effect of soil type and irrigation intervals on plant growth, essential oil yield and constituents of Thymus vulgaris plant. *Am. Euras. J. Agric. & Environ. Sci.*, 4(4), 443-450.

Bhatla, S.C. (2018) Secondary metabolites. In: Bhatla SC, Lal MA (eds) Plant physiology, development and metabolism. Springer, Singapore, pp 1099–1166.

Basavaraja, P.K., Yogendra, N.D., Zodape, S.T., Prakash, R. & Ghosh, A. (2018) Effect of seaweed sap as foliar spray on growth and yield of hybrid maize. *Journal of Plant Nutrition*, 41(14), 1851-1861.

Bayrak, A. & Korkut M.H. (1995) Bazı Tohum Baharatların (Umbelliferae) Yağ Asidi Kompozisyonu ve Özellikle Petroselinik Asit Miktarları Üzerinde Araştırmalar-II. *Standart Dergisi*, 398, 24-32.

Beyzi, E. & Gunes, A. (2017) The effects of boron application on essential oil components of coriander plant (*Coriandrum sativum* L.). *Journal of Agricultural Faculty of Gaziosmanpasa University*, 34(1), 146-152.

Crouch, I.J. & Van Staden, J. (1992) Effect of seaweed concentrate on the establishment and yield of greenhouse tomato plants. *Journal of Applied Phycology*, 4, 291-296.

Crouch, I.J. & Van Staden, J. (1994) Commercial seaweed products as biostimulants in horticulture. *Journal of Home Consumer Horticulture,* 1, 19-76.

Delaquis, P.J., Stanich, K., Girard, B. & Mazza, G. (2002) Antimicrobial activity of individual and mixed fractions of dill, cilantro, coriander and eucalyptus essential oils. *International Journal of Food Microbiology*, 74(1-2), 101-109.

Devi, N.L. & Mani, S. (2015) Effect of seaweed saps Kappaphycus alvarezii and Gracilaria on growth, yield and quality of rice. *Indian Journal of Science Technology*, 8(19), 1-6.

Diederichsen, A. (1996) Coriander (Coriandrum sativum L.): Promoting the conservation and use of underutilized and neglected crops. 3. Institute of Plant Genetics and Crop Plant Research, Gatersleben/International Plant Genetic Resources Institute, Rome.

Featonby-Smith, B.C. & Van Staden, J. (1984) The effect of seaweed concentrate and fertilizer on growth and the endogenous cytokinin content of *Phaseolus vulgaris*. *South African Journal of Botany,* 3, 375-379.

Garg V.K. (2007) Effect of nonsymbiotic microbial inoculants on growth, yield and quality of fennel (Foeniculum vulgare Mill.) grown in sodic soil. *Journal of Spices and Aromatic Crops*, 16(2), 93-98.

Gharib, F.A., Moussa, L.A. & Massoud, O.N. (2008) Effect of compost and bio-fertilizers on growth, yield and essential oil of sweet marjoram (Majorana hortensis) plant. *International Journal of Agriculture Biology*, 10(4), 381-387.

Gokduman, G.I. & Telci, I. (2018) Determination of Yield and Quality Characteristics of Some Coriander (Coriandrum sativum L.) Genotypes in Isparta Conditions. Süleyman Demirel University Journal of Natural and Applied Sciences, 22(3), 1165-1168.

Gucuk, F. (2014) Determination of agronomic and quality properties of coriander (Coriandrum sativum L.) varieties and hats sowed in winter and spring periods under Tokat Kazova conditions. Gaziosmanpasa University Graduate School of Natural and Applied Sciences Department of Field Crops, Master of Thesis, Tokat, 57 s.

Hadian, J., Ebrahimi, S.N., Akramian, M. & Mumivand, H. (2012) Variability in the essential oil content and composition of Iranian landraces of coriander (*Coriandrum sativum* L.), cultivated in a common environment. *Journal of Essential Oil Bearing Plants***,** 15(1), 89-96.

Hernández-Herrera, R.M., Virgen-Calleros, G., Ruiz-López, M., Zañudo-Hernández, J., Délano-Frier, J.P. & SánchezHernández, C. (2014) Extracts from green and brown seaweeds protect tomato (Solanum lycopersicum) against the necrotrophic fungus Alternaria solani. *Journal Applied Phycology*, 26(3), 1607-1614.

Holdt, S.L. & Kraan, S. (2011) Bioactive compounds in seaweed: functional food applications and legislation. *Journal of Applied Phycology*, 23, 543-597.

Hong, Y.P., Chen, C.C., Cheng, H.L. & Lin, C.H. (1995) Analysis of auxin and cytokinin activity of commercial aqueous seaweed extract. *Die Gartenbauwissenschaft*, 60(4), 191-194.

Inan, M., Kirici, S., Giray, E.S., Turk, M. & Taghikhani, H. (2014). Determination of suitable coriander (Coriandrum sativum L.) cultivars for eastern mediterranean region. *Turkish Journal of Field Crops*, 19(1), 1-6.

Izgi, M.N. (2020). Effects of nitrogen fertilization on coriander (*Coriandrum sativum* L.): yield and quality characteristics. *Applied Ecology and Environmental Research*, 18(5),7323-7336.

Jeshni, M.G., Mousavinik, M., Khammari, I. & Rahimi, M. (2017) The changes of yield and essential oil components of German Chamomile (*Matricaria recutita* L.) under application of phosphorus and zinc fertilizers and drought stress conditions. *Journal of the Saudi Society Agricultural Sciences*, 16(1), 60–65.

Jhariya, S. & Jain, A. (2017) Effect of integrated nutrient management on essential oil (volatile oil) of coriander (Coriandrum sativum L.). *International Journal of Current Research Review*, 9(17), 19-25.

Kaplan, G. & Cosge Senkal B. (2021) Farklı fosfor dozlarının kişniş (Coriandrum sativum L.) bitkisinde verim, verim özellikleri ve uçucu yağ oranı üzerine etkileri. *Akademik Ziraat Dergisi*, 10(2), 371-378.

Kandemir, K. (2010) Farklı azot dozu ve sıra aralığının kişnişin verim ve verim unsurları üzerine etkisi. Yüksek lisans tezi, Ordu Üniversitesi Fen Bilimleri Enstitüsü, Ordu.

Khan, W., Rayirath, U.P., Subramanian, S., Jithesh, M.N., Rayorath, P., Hodges, D.M., Critchley, A.T., Craigie, J.S., Norrie, J. & Prithiviraj, B. (2009) Seaweed extracts as biostimulants of plant growth and development. *Journal of Plant Growth* Regulation, 28, 386-399.

Khalid, K.A., & Ahmed, A.M.A. (2021) Effect of Soil Type on Grapefruit and Shaddock Essential Oils. *Journal of Soil Science and Plant Nutrition*, 21(3), 2048-2056.

Karthikeyan, K. & Shanmugam, M. (2016) Development of a protocol for the application of commercial bio-stimulant manufactured from Kappaphycus alvarezii in selected vegetable crops. *Journal of Experimental Biology and Agricultural Sciences*, 4 (1), 92-102.

Kara, M., Soylu, S., Turkmen, M. & Kaya, D.A. (2020) Determination and antifungal activities of laurel and fennel essential oils against fungal disease agents of cypress seedlings. *Journal of Tekirdag Agricultural Faculty*, 17, 264-275.

Kassu, K.T., Dawit, H.H., Wubengeda, A.Y., Almaz, A.T. & Asrat, M.T. (2018) Yield and yield components of coriander under different sowing dates and seed rates in tropical environment. *Advances in Horticultural Sciences*, 32(2), 193-203.

Kaya, N., Yilmaz, G. & Telci, I. (2000) Agronomic and Technological Properties of Coriander (*Coriandrum sativum* L.) Populations Planted on Different Dates. *Turkish Journal of Agriculture and Foresty*, 24,355-364.

Kusuma, M.V., Venkatesha, J., Ganghadarappa, P.M., Hiremath, J.S., Mastiholi, A.B. & Manjunatha, G. (2019) Effect of Integrated Nutrient Management on Growth and Yield of Fennel (*Foeniculum vulgare* Mill.) *International Journal of Current Microbiology and Applied Sciences*, 8(1), 2782-2794.

Laribi, B, Kouki, K, M’hamdi, M, & Bettaieb, T. (2015) Coriander (Coriandrum sativum L.) and its bioactive constituents. *Fitoterapia*, 103, 9-26.

Momin, A.H., Acharya, S.S. & Gajjar, V. (2012) Coriandrum sativum - review of advances in phytopharmacology. *International Journal of Pharmaceutical Sciences and Research*, 3, 1233-1239.

Mancuso, S., Azzarello, E., Mugnai, S. & Briand, X. (2006). Marine bioactive substances (IPA extract) improve foliar ion uptake and water stress tolerance in potted Vitis vinifera plants. *Advances Horticultural Sciences*, 20, 156-161.

Nadeem, M., Anjum, F.M., Khan, M.I., Tehseen, S., El-Ghorab, A. & Sultan, J.I. (2013) Nutritional and medicinal aspects of coriander (Coriandrum sativum L.)A review. *British Food Journal*, 115(5), 743-755.

Nguyen, D.T.P., Kitayama, M., Lu, N. & Takagaki, M. (2020) Improving secondary metabolite accumulation, mineral content, and growth of coriander (Coriandrum sativum L.) by regulating light quality in a plant factory. *The Journal of Horticultural Science and Biotechnology*, 95(3), 356-363.

Ozel, A., Guler, G. & Erden, K. (2009) Effect of different sowing time on yield and some plant characters of coriander (Coriandrum sativum L.) under the Harran plain conditions. *Journal of the Faculty of Agriculture*, 13(4), 41-48.

Ozyazıcı, G. (2021). Influence of Organic and Inorganic Fertilizers on Coriander (Coriandrum sativum L.) Agronomic Traits, Essential Oil and Components under Semi-Arid Climate. *Agronomy*, 11, 1427.

Palaniappan SP, Annadurai K (1999). Organic Farming Theory & Practice. Scientific Publishers (India) 5A, New Pali Road, P.O. Box 91 Jodhpur 342001(India).

Panda, D., Pramanik, K. & Nayak, B.R. (2012) Use of Sea Weed Extracts as Plant Growth Regulators for Sustainable Agriculture. International Journal of Bio-resource and Stress Management, 3(3), 404-411.

Pooja, P.K. & Nagre Yadav, H. (2017) Influence of different levels of nitrogen and phosphorus on seed yield and economics of coriander (*Coriandrum sativum* L.). *Journal Pharmacognosy and Phytochemistry*, 6(5), 157-160.

Rathore, S.S., Chaudhary, D.R., Boricha, G.N., Ghosh, A., Bhatt, B.P., Zodape, S.T., & Patolia, J.S. (2009) Effect of seaweed extract on the growth, yield and nutrient uptake of soybean (Glycine max) under rainfed conditions. *South African Journal of Botany*, 75, 351-355.

Ravi, R., Prakash, M. & Bhat, K.K. (2007) Aroma characterization of coriander (Coriandrum sativum L) oil samples. *Europen Food Ressearch and Technology*, 225(3-4), 367-374.

Roussos, P., Denaxa, N. & Damvakaris, T. (2009) **Strawberry fruit quality attributes after application of plant growth stimulating compounds.** *Scientia Horticulturae*, 119(2), 138-146.

Said-Al Ahl, H.A.H., Sabra, A.S., Alataway, A., Astatkie, T., Mahmoud, A.A. & Bloem, E. (2019) Biomass production and essential oil composition of Thymus vulgaris in response to water stress and harvest time*. Journal of Essential Oil Research*, 31(1), 63-68.

Spinelli, F., Fiori, G., Noferini, M., Sprocatti, M. & Costa, G. (2009) Perspectives on the use of a seaweed extract to moderate the negative effects of alternate bearing in apple trees. Journal of Horticultural Science Biotechnology, 84, 131-137.

Suman, P., Lakshminarayana, D., Prasanth, P. & Naik, S. (2019) Effect of integrated nutrient[management on yield parameters of coriander (Coriandrum sativum L.) cultivars under](https://zambrut.com/integrated-nutrient/) Telangana conditions. *International Journal of Chemical Studies*, 7(1), 392-394.

Tekkanat A. & Soylu S. (2005) Determination of important agronomical characters in popcorn cultivars, *Journal of Selcuk University Faculty of Agriculture*, 19(37), 51-60.

Telci, I., Bayram, E. & Avcı, B. (2006a) Changes in yields, essential oil and linalool contents of Coriandrum sativum varieties (var. vulgare Alef. and var. microcarpum DC.) harvested at different development stages. *European Journal of Horticultural Science*, 71, 267-271.

Telci, I., Toncer, O.G. & Sahbaz, N. (2006b) Yield, essential oil content and composition of Coriandrum sativum varieties (var. vulgare Alef and var. microcarpum DC.) grown in two different locations. *Journal of Essential Oil Research*, 18, 189-193.

Telci, I., Demirtas, I., Bayram, E., Arabacı, O. & Kacar, O. (2010) Environmental variation on aroma components of pulegone/piperitone rich spearmint (*Mentha spicata* L.). *Industrial Crops and Products*, 32, 588-592.

Telci, I, & Hisil, Y. (2008) Biomass yield and herb essential oil characters at different harvest stages of spring and autumn sown Coriandrum sativum. *European Journal of Horticultural Science*, 73(6), 267-272.

Toncer O., Tansı L.S. & Kızıl S. (1998) The Effect of Different Sowing Time on Essential Oil of Coriander in Gap Region. *Anadolu Journal of the Aegean Agricultural Research Institute*, 8(2), 101-105.

Verma, V.K., Sehgal, O.P. & Shiman, S.R. (2000) Effect of nitrogen and GA3 on Carnation. *Journal of Ornamental Horticulture (New series)*, 3(1), 64.
