# Supplementary material for: Effect of foliar application of seaweed (organic fertilizer) on yield, essential oil and chemical composition of coriander
Source: PLoS One. 2022 Jun 2;17(6):e0269067. doi: 10.1371/journal.pone.0269067 (PMC9162348; doi:10.1371/journal.pone.0269067)
Supplement: Attachment — Submitted filename: PONE-D-22-06119 comments.docx [file pone.0269067.s002.docx]

Thank you for the opportunity to review this manuscript. The investigation is useful but the study itself suffers from some conceptual flaws, which are noted in more detail in the attached review

The manuscript needs extensive revision for language and grammar.

As a first step, I advise the authors to find a native English speaker to proofread the manuscript.

**Title**

The title is good, but you can use a more attention-grabbing title.

**Abstract**

The abstract should provide a concise and complete summary of the manuscript. ‘‘missed’’.

### I am missing the key findings of the study.

I am missing the application and recommendation of the outcomes.

There are important aspects to review regarding some grammar and English usage issues.

The highlights are general, please write more specific

**Introduction**

Recent references must be cited.

Be careful about citing old references. The rule of thumb is to go back at most five to six years.

Line 45-46 this information isn't important, you can delete it.

Line 48-51 Reference is needed

Line 51-53 please avoid repetition.

Line 58 recent references should be cited

Line 91 should start a new paragraph.

Lines 96-99 Please rewrite this sentence to make it more clear and understandable. ‘‘For this reason, this study was carried out to determine the changes on yield, quality and compounds of essential oils by applying in different doses and at different times (before or initial stage of flowering) of seaweed to two different coriander varieties.’’

In your manuscript, you must include clear research question, aim, and objectives.

What is the significance of your research?

**Materials and methods**

**Methodology section have major limitation, should be more clear**

Line 104 ‘tests’ is not appropriate word

Table 2. Main climate data of the research site for the long term (1981–2019) **OR** 1929-2020

Lines 135-138 confused, please rewrite.

Line 141 Raw Protein Ratio, confused

Line 142 ‘the Dumas method’ reference is needed

Line 155 Extraction of essential oils

Line 155 ‘Reference is needed’

Line 173 The data were under went to statistical analysis?????

The language is ambiguous and confused.

**Results and Discussion**

**Again**, I advise the authors to find a native English speaker to proofread the manuscript.

It is better to separate the results and the discussion.

This section can be improved. Please present your key findings in an orderly and logical sequence, without interpretation.

Lines 179-180 ‘‘Seaweed fertilizer was applied on the plants 40th and 60th days after sowing and it was applied with different dosages’’. It should be mentioned in the materials section rather than here.

Lin 324: Nutrients of Volatile Oils????????

Lin 186 ‘‘important’’ is not appropriate word should be ‘‘significant’’.

**Discussion**

The section is poor. The purpose of the discussion section is to interpret and describe the significance of your findings in relation to what was already known.

Recent references must be included in the manuscript.

<https://doi.org/10.1016/j.indcrop.2020.113202>

**Conclusion**

The conclusion should summarize the paper's findings and generalize their importance, discuss ambiguous data, and recommend further studies.

This chapter is more like discussion part. Please revise and strengthen this chapter and provide conclusions and recommendations based on the results of your research**.**

Some editing for English language is required throughout the manuscript due to too many mistakes such as:

**Lin**e **366: ‘‘locationswere’’** change to locations were

Line 371: mora???

Line 377 wafer???

**References**

Be careful about citing old references. The rule of thumb is to go back at most five to six years.

**Best wishes**
