# Supplementary material for: Effect of foliar application of seaweed (organic fertilizer) on yield, essential oil and chemical composition of coriander
Source: PLoS One. 2022 Jun 2;17(6):e0269067. doi: 10.1371/journal.pone.0269067 (PMC9162348; doi:10.1371/journal.pone.0269067)
Supplement: Attachment — Submitted filename: PONE-D-22-06119 response to reviewers.docx [file pone.0269067.s003.docx]

Dear Editor,

Thank you for arranging a review of the submission. The manuscript is now carefully revised considering all the comments from the two anonymous reviewers and the editor. Please find in following a response to each of the question/suggestion/comment from the reviewers. In addition, references are given by numbering according to the journal style. Thank you for your time and input.

Regards,

**Comments from the Reviewers**

**Reviewer 1**

Comment: The author proceeds with the use of an "algae-based" fertilizer, which is why it was supposed to have indicated its origin: brand, composition, etc.The author limited his characterization to this sentence: "The seaweed applied fertilizer which has 10% organic compounds, 0.4% alginic acid"... so it could have any composition, as it does not specify which algae and which production methods are used. behind the production of this "algae-based" fertilizer.The presence of alginic acid in its composition does not make it a fertilizer based on algae, but based on "alginic acid".The author will have to redo this part of the manuscript, as it is essential to understand its effect on the production of the aromatic plant.

Response: The trade name, company and content of the seaweed are given in the material and method.

**Reviewer 2**

Thank you for the opportunity to review this manuscript. The investigation is useful but the study itself suffers from some conceptual flaws, which are noted in more detail in the attached review

The manuscript needs extensive revision for language and grammar.

**Comment:** As a first step, I advise the authors to find a native English speaker to proofread the manuscript.

Response: You are right, there were language related issues in the manuscripts, which have been resolved now after a thorough check and corrections done by an English language expert.

**Comment: Title**

The title is good, but you can use a more attention-grabbing title.

Response: The title has been improved now.

**Abstract**

**Comment:** The abstract should provide a concise and complete summary of the manuscript. ‘‘missed’’.

Response: The abstract has been revised now.

### Comment: I am missing the key findings of the study.

### Response: Final findings have been added now.

**Comment:** I am missing the application and recommendation of the outcomes.

Response: In the last sentence of the abstract, the final recommendation and outcomes have been added.

**Comment:** There are important aspects to review regarding some grammar and English usage issues.

Response: Grammar and English have been rechecked and corrected.

**Comment:** The highlights are general, please write more specific

## Response: This part is not given because there are no highlights in the journal's Style and Format.

**Introduction**

**Comment:** Recent references must be cited.

Response: References have been updated by the recently available literature.

**Comment:** Be careful about citing old references. The rule of thumb is to go back at most five to six years.

Response: Old references have been removed or updated with recently available references.

**Comment:** Line 45-46 this information isn't important, you can delete it.

Response: This information has been deleted.

**Comment:** Line 48-51 Reference is needed

Response: Reference (du Jardin, 2015) has been added now.

**Comment:** Line 51-53 please avoid repetition.

Response: Repeated sentence has been changed now.

**Comment:** Line 58 recent references should be cited

Response: Replaced with new recent reference (Ali et al., 2019, 2021).

**Comment:** Line 91 should start a new paragraph.

Response: Thanks, followed accordingly.

**Comment:** Lines 96-99 Please rewrite this sentence to make it more clear and understandable. ‘‘For this reason, this study was carried out to determine the changes on yield, quality and compounds of essential oils by applying in different doses and at different times (before or initial stage of flowering) of seaweed to two different coriander varieties.’’

Response: This sentence has been revised now to improve its meaning and clarity.

**Comment:** In your manuscript, you must include clear research question, aim, and objectives.

Response: Aims and objectives have been clearly mentioned now in the end of the Introduction section.

**Comment:** What is the significance of your research?

Response: In the study, for the first time, seaweed, an organic fertilizer, was applied to coriander and significant yield increases were obtained.

**Materials and methods**

**Comment: Methodology section have major limitation, should be more clear**

Response: The methodology is **revised to make it clear as much as possible.**

**Comment:** Line 104 ‘tests’ is not appropriate word

Response: This word has been replaced with anothersuitable word “experiments”.

**Comment:** Table 2.Main climate data of the research site for the long term (1981–2019) **OR** 1929-2020

Response: Sorry about this mistake, the data is for 1929-2020, this is now corrected in the table heading.

**Comment:** Lines 135-138 confused, please rewrite.

Response: This line has been revised now.

**Comment:** Line 141 Raw Protein Ratio, confused

Response: Now we use “Crude protein content (%)” throughout the manuscript.

**Comment:** Line 142 ‘the Dumas method’ reference is needed

Response: Reference (Saint-Denis and Goupy, 2004)is added now.

**Comment:** Line 155 Extraction of essential oils

Response: Thanks, the word ‘isolation’ has been changed to ‘extraction’.

**Comment:** Line 155 ‘Reference is needed’

Response: Reference (Turkment and Mert, 2020) has been added.

**Comment:** Line 173 The data were under went to statistical analysis?????

Response: Yes, the data were applied with a statistical analysis, details are provided at end of the Materials and Methods section.

**Comment:** The language is ambiguous and confused.

Response: Whole of the manuscript has been revised thoroughly.

**Results and Discussion**

**Comment: Again**, I advise the authors to find a native English speaker to proofread the manuscript.

Response: The manuscript has now been rechecked and a language expert corrected the language mistakes.

**Comment:** It is better to separate the results and the discussion.

Response: We think that the combine presentation of the results and discussion is more suitable, hence we request to keeping this format.

**Comment:** This section can be improved. Please present your key findings in an orderly and logical sequence, without interpretation.

Response: Thanks, the section has been revised now as per the comment.

**Comment:** Lines 179-180 ‘‘Seaweed fertilizer was applied on the plants 40th and 60th days after sowing and it was applied with different dosages’’. It should be mentioned in the materials section rather than here.

Response: Thanks, now it is mentioned in the materials and method section.

**Comment:** Lin 324: Nutrients of Volatile Oils????????

Response: It is essential oil contents and has been updated in the whole manuscript.

**Comment:** Lin 186 ‘‘important’’ is not appropriate word should be ‘‘significant’’.

Response: Thanks, it has been replaced now.

**Discussion**

**Comment:** The section is poor. The purpose of the discussion section is to interpret and describe the significance of your findings in relation to what was already known.

Response: The discussion portion has been properly revised now.

**Comment:** Recent references must be included in the manuscript.

<https://doi.org/10.1016/j.indcrop.2020.113202>

Response: Thanks, now we have tried to update the old references.

**Comment: Conclusion**

The conclusion should summarize the paper's findings and generalize their importance, discuss ambiguous data, and recommend further studies.

This chapter is more like discussion part. Please revise and strengthen this chapter and provide conclusions and recommendations based on the results of your research**.**

Response: This chapter has been properly revised now.

**Comments:**Some editing for English language is required throughout the manuscript due to too many mistakes such as:

**Response: Sorry you had to bother with the language mistakes, these are now removed through a** thorough check and corrections done by an English language expert.

**Comments: Lin**e **366: ‘‘locationswere’’** change to locations were

Response: Thanks, revised.

**Comments:**Line 371: mora???

Response: It is “more" and now has been changed in the manuscript.

**Comments:**Line 377 wafer???

Response: It’s“after” and has been changed now accordingly.

**Comments: References**

Be careful about citing old references. The rule of thumb is to go back at most five to six years.

Response: Old references have been removed or updated with recently available references.

**Best wishes**
